# Study on Fenton-based discoloration of reactive-dyed waste cotton prior to textile recycling

**DOI:** 10.1038/s41598-024-75450-w

**Published:** 2024-10-19

**Authors:** Elise Meurs, Mohammad Neaz Morshed, May Kahoush, Nawar Kadi

**Affiliations:** 1 Department of Textile Technology, The Swedish School of Textiles, Faculty of Textiles, Engineering and Business, University of Borås, Allégatan 1, 503 32 Borås, Sweden; 2 Department of Engineering and Chemical Sciences, Karlstad University, Universitetsgatan 2, 651 88 Karlstad, Sweden

**Keywords:** Cotton, Discoloration, Fenton-oxidation, Reactive dye, Textile recycling, Pollution remediation, Environmental impact

## Abstract

The aim of this study is to investigate the feasibility of an alternative Fenton-based advanced oxidation process for the discoloration of reactive-dyed waste cotton as a pre-treatment for textile recycling. For that, pre-wetted dark-colored (black and blue) knitted samples of 300 cm^2^ are treated in 1200mL Fenton-solution containing 14 mM Fe^2+^ and 280mM H_2_O_2_ at 40 °C. Characterization of the textiles before and after the treatments are performed by UV VIS-spectrophotometry measuring color strength, microscopy, FTIR spectroscopy, thermal analysis and tensile testing measuring tenacity and elongation. Afterwards, the cotton is mechanically shredded for qualitative analysis of the recyclability. The color-strength measurements of the black and blue cotton led to discoloration-efficiencies of respectively 61.5 and 72.9%. Microscopic analysis of discolored textile fabric also showed significant fading of the colored textiles. Mechanical analysis resulted in reduced tensile strength after treatment, indicating oxidation of the cellulosic structure besides the degradation of the dye-molecules, also confirmed by reductions in thermal stability found after thermal analysis. Shredding of the fabric resulted in enhanced opening, but shorter remaining fibers after treatment. The findings of this study provide a proof-of-concept for an alternative color-stripping treatment concerning a Fenton-based advanced oxidation process as a pre-treatment for textile recycling.

## Introduction

Changes in our consumption behaviour and the development of fast-fashion have caused an increasing trend of the annual global production of textiles^1^ and therefore also increasing amounts of landfill. Considering the industry’s high energy- and water-demand, the textile sector contributes significantly to environmental pollution and resource utilization^2^. In order to decrease the environmental impact of the textile industry, waste-streams in the shape of landfill need to be diminished by efficient textile recycling of both post-production and post-consumer waste textiles. For example mechanical recycling and pulping allows for the reuse of waste cotton in fiber to fiber (F2F) recycling of textile waste, increasing the circularity and decreasing the amount of waste-production and landfill while minimizing the request for raw material, which would indicate lower agricultural needs (lower demand for land-occupation, water and chemicals)^3^.

However, efficient textile-recycling is often still difficult due to contaminating substances such as dyes and finishes which are frequently added during various processing-treatments^3^. Therefore, preparatory treatments such as color-stripping are important for obtaining increased recycling efficiency, producing regained textiles which meet the quality standards for its reuse in the textile-industry^4^.

Several approaches for the discoloration of cotton already exist. Since reactive dyes bind covalently to the textile, a method specifically degrading the dye-molecule or cleaving the covalent bond is needed for discoloration^3,5^. The most common industrial processes consist of chemical treatments using alkaline reductive processes which often require high concentrations of various chemical agents such as KMnO_4_, Na_2_S_2_O_4_ and H_2_O_2_^6,7^. However, these agents are known to be damaging to the textile-material and toxic to the environment. Furthermore, the processes require high temperature- and liquor-consumption, resulting in a pre-treatment for textile recycling with high environmental impact^6–8^. For example, activated alkaline hydrogen peroxide treatments require temperatures of 70–90 °C and bleaching-solutions with 0.5–1M H_2_O_2_ at pH ≈ 10 for 60–180 min in order to obtain discoloration percentages of around 50–96%^9,10^, thus requiring high amounts of energy and the highly reactive and toxic chemical H_2_O_2_^11^. In respect of the current toxic industrial processes, alternative oxidative processes^12,13^ and biological processes (using enzymes)^14,15^ have recently become frequent research topics, aiming to increase the sustainability of color-stripping treatments of waste-textiles.

The biological processes using micro-organisms are however challenged by the varying degrees of effectiveness across different dye-classes due to the specificity of the enzymatic reactions. Also, difficulties in achieving optimal operation conditions concerning temperature, pH and substrate concentration increase the challenges for its use in large scale processes^14^.

Also, alternative chemical processes have been investigated as environmental friendly substitutes of current industrial color-stripping treatments. Advanced oxidation processes (AOP) have acquired interest in research for the oxidation of a diverse range of molecules, including complex textile dyes^12,13^. The AOP-group consists of different mechanisms (with the most common processes being photocatalytic-oxidation, ozone-oxidation and Fenton-oxidation)^16,17^, all generating the highly reactive hydroxyl radicals (OH* with oxidation potential ${\text{E}}_{OH*}^{^\circ }$ = 2.8V compared to ${\text{E}}_{{H}_{2}{O}_{2}}^{^\circ }$ = 1.78V)^16,18^, in which the formation of the radicals can also be enhanced through addition of energy from ultrasound- and/or UV-radiation^16,17^.

Considering the use of AOP’s for the degradation of dye-molecules, both photocatalytic- and ozone-oxidation have already been investigated in research towards discoloration of waste-water^12,19,20^ and waste-textiles^6,21,22^. Fenton and Fenton-like mechanisms have been shown to be a versatile tool in the degradation of organic pollutants for waste-water treatments, for example in the context of discoloration of waste-water (dye in solution)^13,15,23–32^. Table 1 gives an overview of the state of the art of research on Fenton and Fenton-like discoloration of dye-solutions by listing examples of references, together with the range of optimal efficiency of the treatments obtained in these references, given as a measure of COD-removal. More review-articles on Fenton-based waste-water treatments^18,33–35^ and studies on degradation of dye in solution using Fenton-like mechanisms^36–38^ can be found in literature. Nonetheless, to the best of our knowledge, the use of Fenton-oxidation for discoloration of fabric has not yet been studied.

**Table 1:** Overview of state of the art of Fenton-oxidation of dye in solution.

Discoloration-process of dye-solution	Obtained optimal COD removal	Examples of references in literature
Fenton	80–99.3%	Swaminathan et al.^23^, Sundararaman et al.^25^, Adar^39^, Dobrosz-Gómez et al.^27^
Electro-Fenton	75.2–99.9%	Huang et al.^13^, Malakootian et al.^28^, Özcan & Özcan^24^
Photo-Fenton	88–98%	Chacón^29^, Huang et al.^13^, Sundararaman et al.^25^
Sono-Fenton	51–82%	Sundararaman et al.^25^, Zhang et al.^30^, Özdemir^31^
Bio-Fenton	78–90%	Karimi et al.^15^, Eskandarian et al.^32^, Morshed^26^

The reactions generally occurring in the Fenton-mechanism are given in reaction 1–5^34,40^. The Fe(II)-cation is oxidized while generating hydroxyl-radicals (reaction 1), which will later act as the main oxidizing agent in the degradation-process of the organic substances^40^ as is visible in reaction 2 and 3, where the highly oxidative OH-radicals attack unsaturated bonds in the organic substance (R). In theory, this organic substance can be completely degraded into CO_2_ and H_2_O^26^, as in reaction 3. The oxidized Fe-cation (Fe^3^^+^) will have to react with the peroxide in order to regenerate the Fe-catalyst for continuation of the mechanism (reaction 4 and 5). However, this regeneration occurs at a much lower speed (k_1_ = 40–80 L/mol*s compared to k_4_ = 9*10^–7^ L/mol*s) and is therefore the rate-determining step of the mechanism^40^.

1 $${Fe}^{2+}+{H}_{2}{O}_{2}\to {Fe}^{3+}+ {OH}^{-}+{OH}^{*}$$

2 $$R+{OH}^{*}\to {R}_{oxidized}+{H}_{2}O$$

3 $${R}_{oxidized}+{OH}^{*}\to {CO}_{2}+{H}_{2}O$$

4 $${Fe}^{3+}+{H}_{2}{O}_{2}\to {Fe}^{2+}+{H{O}_{2}}^{*}$$

5 $${Fe}^{3+}+{H{O}_{2}}^{*}\to {{Fe}^{2+}+ O}_{2}+{H}^{+}$$

The performance of the mechanism depends on several process-parameters such as pH, concentrations of chemical agents, temperature and reaction time. For example, the pH determines the ability of the ferrous cation to regenerate; with increasing pH (starting from pH ≈ 4), Fe^3+^ will form a brown-colored precipitate Fe(OH)_3_ which is not able to contribute to the oxidation of the organic molecules. Therefore, the Fenton-oxidation works most efficient in a low pH^40^. However, at too low pH (≤ 2), the hydroxyl-radicals can get protonated, also leading to a decreased efficiency^41^. The optimal pH-range for Fenton-reactions thus lies between pH 2.5–4^40,41^. Based on the mechanism, some other efficiency-determining process-parameters are the Fe^2+^ and H_2_O_2_ concentration, with an increasing trend in efficiency with increasing concentrations which stagnates after a certain level^41,42^. For the iron-source, the stagnation is caused by increasing concentration of Fe^3+^ due to the slow reconversion to the reactive ferrous form and consequently more chance at undesired precipitation-reactions into inactive Fe(OH)_3_^23,42^. For the H_2_O_2_-source, the stagnation is caused by the instability of the chemical agent, which causes consumption of the formed hydroxyl-radicals with production of hydroperoxyl-radicals (${{\text{HO}}_{2}}^{*}$) with lower oxidation potential (${\text{E}}_{{HO}_{2}}^{^\circ }$= 1.7V compared to ${\text{E}}_{\text{OH}}^{^\circ }$ = 2.8V) and possibly even consumption in recombination reactions^23,42^. Considering that this Fenton-oxidation mechanism is endothermic, increased temperatures will give higher conversion percentages^41,43^. However, with an eye on sustainability, too high energy-consumptions due to high temperatures should be avoided, and literature mostly considers temperatures between 35 and 45 °C^41,43^. When considering the reaction-time, the treatment-efficiency increases with longer reaction-duration until, at a certain point, most of the hydrogen-peroxide will be consumed and the reaction will slow down^23^. Table 2 gives an overview of optimal conditions for Fenton-treatments of dye in solution (at $\sim$ 20mg/L dye).

**Table 2:** Overview of optimal conditions for Fenton-discoloration of dye in solution ($\sim$ 20mg/L dye), based on literature-sources^23,41,42^.

Parameter	pH	T (°C)	[H_2_O_2_] (mM)	[Fe^2+^]/[H_2_O_2_]	Time (min)
Optimal value	2.5–4	35–45	40–70	0.05	30–120

Despite the extensive research-sources on the use of Fenton for waste-water-treatments, the use of Fenton-oxidation for discoloration of fabric forms a novel research-topic. Yet, successful thorough investigation of this discoloration-process and investigation of the applicability on industrial scale could allow for recycling pre-treatments with lower environmental impact, while increasing the efficiency of the recycling process itself. This would allow increased efficiency and scale of the recycling process, and hereby reducing the amount of landfill and increasing the circularity of materials. Therefore, the search for efficient and environmental friendly decolorizing processes plays an important role in the reduction of pollution caused by the textile industry.

As a first step in this investigation towards the use of Fenton-based advanced oxidation as a tool for color-stripping of cotton as a preparatory treatment prior to textile recycling, this research article aims to analyze the feasibility of the mechanism in the scope of textile discoloration. The influence of the treatment on the mechanical and thermal properties of the fabric and the mechanical recycling process afterwards will be assessed.

## Experimental

### Materials

For the Fenton-processes, analytical-grade iron(II)sulfate hepta-hydrate (FeSO_4_ × 7H_2_O, $\ge$ 99.0% purity) and hydrogen peroxide solution (H_2_O_2_, 35 wt%) were purchased from Sigma Aldrich and Merck respectively. Two different reactive dyes; Reactive Blue 19 and Remazol Black B (C.I. Reactive Black 5) were purchased from Zenit and DyStar respectively for the preparation of simulated dark colored waste textile fabric, made from raw cotton yarn (24Nm) purchased from Stiafilco. Sodium chloride (NaCl), sodium carbonate (Na_2_CO_3_) and all relevant basic chemicals for this study were purchased from Sigma Aldrich and used as its original form without further purifications. Deionized water from the Milli-Q® Direct 8 water-purification system installed at the research lab of Borås University was used throughout all experiments.

### Preparation of dark colored cotton fabric

For the preparation of simulated dark colored waste textile fabric, knitted cotton fabric (with a gauge of 20 needles per inch) was dyed with a black and blue dye using a two-step reactive dyeing method in a Pyrotec MB2-machine. In a first step, the cotton is treated with 6% omf dyestuff and electrolyte-concentration (NaCl) of 80 g/L, in a liquor ratio of 1:10 for the black dye and liquor ratio of 1:25 for the blue dye—as recommended by the technical data-sheets of the suppliers. This step is applied for 30 min at 25 °C. In a second step, the temperature is increased to 60 °C at 1 °C/min, after which an alkaline-concentration (Na_2_CO_3_) of 20 g/L is added to increase the pH up to 9–10, and the fabrics are treated in these conditions for 45 min.

Afterwards, the fabrics were neutralized and thoroughly washed. Unfixed dyestuff was removed by placing the dyed cotton in flasks with water in an ultrasound cleaning bath (VWR® USC-TH) for 10 min at 40 °C. This step was repeated until complete removal of unfixed dyestuff from the freshly prepared fabric was achieved. The prepared fabrics were used as reference to study the discoloration of colored textile waste using Fenton-based advanced oxidation processes.

### Method of discoloration of colored textile waste using Fenton-based advanced oxidation processes

Only optimal conditions for Fenton-discoloration of waste-water can be found in literature (Table 2). However, in this literature, the dye in solution will be in lower concentrations ($\sim$ 20 mg/L) compared to the dye bound to the cotton fabric used in this study. Consequently, the optimal conditions in Table 2 are likely not yet optimal for the removal of color from fabric. Therefore, a preliminary study was performed to obtain the required concentrations of Fenton-reagents for the discoloration of waste cotton, starting from the optimal conditions for Fenton-treatment of waste-water. The influence of increasing concentration of the Fenton-solution on the efficiency of the discoloration of fabric was investigated by using four different concentrations; the optimal concentrations in Table 2, and multiplying these optimal concentrations by 2, 4 and 8. The conditions used in these four treatments are presented in Table 3. The temperature was chosen to be set at 40 °C. A detailed description on the method and data obtained from the preliminary study can be found in the supplementary document.

**Table 3:** Four different conditions used in pre-tests, for a fabric of 5 × 5 cm in 100 mL solution.

	[Fe^2+^] (mM)	[H_2_O_2_] (mM)	T (°C)
Fenton 1	3.5	70	40
Fenton 2	7	140	40
Fenton 3	14	280	40
Fenton 4	28	560	40

Based on the results from preliminary studies, the following up-scaled conditions were chosen for further Fenton-discoloration of cotton; fabric-samples of 300cm^2^ are treated with Fenton-solutions with 14mM Fe^2+^ and 280mM H_2_O_2_ in 1200mL Fenton-solution (prepared with demineralized water), at a temperature of 40 °C (maintained by means of a warm-water bath), while constantly stirring the solution with a magnetic stirrer at 400 rpm. Due to the use of rather hydrophobic raw-cotton in this study, the samples are pre-wetted in de-ionized water in an ultrasound cleaning bath (VWR® USC-TH) for 10 min at 40 °C. These samples are then treated in Fenton-solution for 60 min, and this treatment is repeated for 3 different samples for each dye. The pH is monitored (but not altered) at the beginning and the end of the reaction by means of a pH-sensor (FiveEasy Standard pH Meter Line, Mettler Toledo).

### Material characterizations

The Kubelka–Munk color strength (K/S at ${\lambda }_{max}$) gives a value of the ratio of light absorbed to the amount of light scattered after radiation of a surface, with increased absorption and reduced scattering leading to higher color strengths^44^. This K/S-value of the cotton fabric before and after discoloration is measured by means of a UV–VIS-spectrophotometer (Datacolor 500) illuminating the sample with a D65/10 light-source, containing a FL40-UV filter (0%UV), and calibrated by comparison of a sample diagnostic tile to a reference diagnostic tile. The measurements are performed on a double folded fabric (4 layers) to avoid contributions from the background material. From the K/S-values before and after the treatment, the discoloration percentage can be calculated according to the following equation:

$$\begin{aligned} & discoloration\% \\ & \quad = \frac{color\ after\ dyeing - color\ after\ Fenton\ treatment}{{color\ after\ dyeing}} \times 100 \\ & \quad = \frac{{\left( {\left( {K/S_{{\lambda_{max} ,dyed}} - K/S_{{\lambda_{max} , raw\ cotton}} } \right) - K/S_{{\lambda_{max} ,Fenton\ treated}} } \right)}}{{\left( {K/S_{{\lambda_{max} ,dyed}} - K/S_{{\lambda_{max} , raw\ cotton}} } \right)}} \times 100 \\ \end{aligned}$$

Images of the unraveled yarns are captured with a reflection-microscope Inskam 315-W 5MP Dual Lens Digital Microscope (Inskam) with magnification of 250×. Images of the cotton fibers are made with a transmission-microscope Eclipse Ei Microscope (Nikon), with a magnification of 400×. All images are acquired through KoPa WiFi Lab software.

Fourier-transform infrared spectroscopy captures the spectra of the raw, dyed and Fenton-treated cotton by means of the FTIR-instrument Nicolet iS10 (Thermo Scientific), with a resolution of 4 cm^−1^ and 64 scans per sample. To calibrate the measurements, the spectrum of the background (ambient air) was made and deducted from following measurements of the fabric-samples. This test is repeated 3 times for the black and blue dye.

Thermogravimetric analysis (TGA) is performed by TGA Q500 (TA Instruments) with samples of ~ 10mg of the dyed and discolored cotton in a platinum pan and an inert N_2_-atmosphere, with a temperature increase from 30 up to 500 °C and a heating rate of 20 °C/min. This test is repeated for 3 different samples. Differential scanning calorimetry (DSC) is performed by DSC Q2000 (TA Instruments) with samples of ~ 5mg which are cooled and heated in three cycles with a heating rate of 10 °C/min and in an inert N_2_-atmosphere: heated from − 25 up to 250 °C, cooled back to − 25 °C, and heated again to 250 °C. This test is repeated for 3 different samples.

The strength, elongation and tenacity of unraveled yarn are measured during stretching with a Tensolab 2512A (Mesdan-Lab), according to the standard for “Determination of single-end breaking force and elongation at break using constant rate of extension (CRE) tester—yarns from packages” (EN ISO 2062). For both the dyed and discolored cotton, this test was repeated for 10 samples of unraveled yarn taken from random locations of the knitted textile-samples. The samples were conditioned in a standard atmosphere for a minimum of 4 h before the testing, according to the standard for “Standard atmospheres for conditioning and testing—textiles” (EN ISO 139).

### Evaluation of F2F mechanical recycling of discolored cotton fabric

For a qualitative study on the potential of mechanical recycling of the discolored cotton, samples of 15cmx20cm are shredded using a Trim Fabric Opening Machine (Kingtech Machinery), while collecting the opened fabric for qualitative analysis of the amount of opened fabric and length of cotton fibers remaining after shredding. The edge trim opener is composed of a tearing cylinder, three working rollers and two feeding rollers. The feeding and working rollers are clothed with rigid wire clothing, while the tearing drum can be clothed with pinned wooden lags or rigid wire. The choice of the wire clothing is subject to the material to be recycled.

### Method of statistical analysis

Two-sided t-tests (paired in case of comparison between dyed and Fenton-treated fabric) are performed with CI = 95% (or $\alpha$ = 0.05). For obtained p-values < $\alpha$  = 0.05, the difference in results can be assumed ‘significant’.

## Results and discussion

Here, two dark-colored cotton samples were studied for discoloration using Fenton-based advanced oxidation processes. Following section will present the analysis of discoloration of black- and blue-colored waste-cotton based on the results obtained.

### Analysis of discoloration of waste cotton fabric

**Fig. 1:**
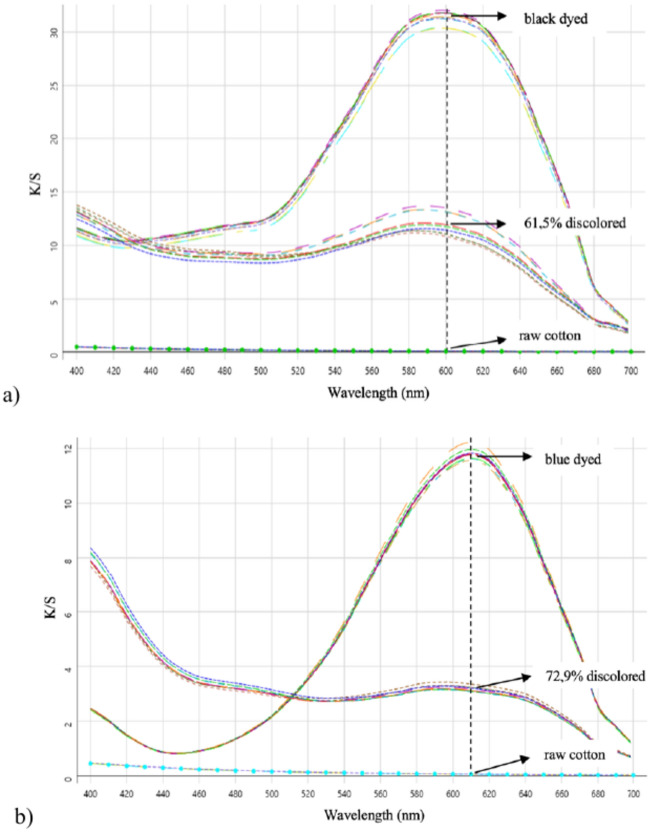
Plots of measured K/S-values of black cotton (**a**) and blue cotton (**b**) before and after Fenton-treatment, compared to the K/S-plot of raw cotton.

From the K/S-plot in Fig. 1a, the black-dyed cotton showed a reduction from an average color strength K/S = 31.30 $\pm$ 0.54 to K/S = 11.99 $\pm$ 1.16 after the treatment, leading to a discoloration of 61.54% considering a mean K/S_raw cotton_ = 0.072. In the K/S-plot in Fig. 1b, the blue-dyed cotton showed a reduction from an average K/S = 11.80 $\pm$ 0.11 to K/S = 3.21 $\pm$ 0.07 after the treatment, leading to a discoloration of 72.90% considering a mean K/S_raw cotton_ = 0.067. Statistical testing of the K/S-values before and after the treatment (with α = 0.05 and n = 3) indicate significant discoloration for both the black- (*p* = 0.003) and blue-dyed cotton (*p*  < 0.001) due to the Fenton-oxidation.

**Fig. 2:**
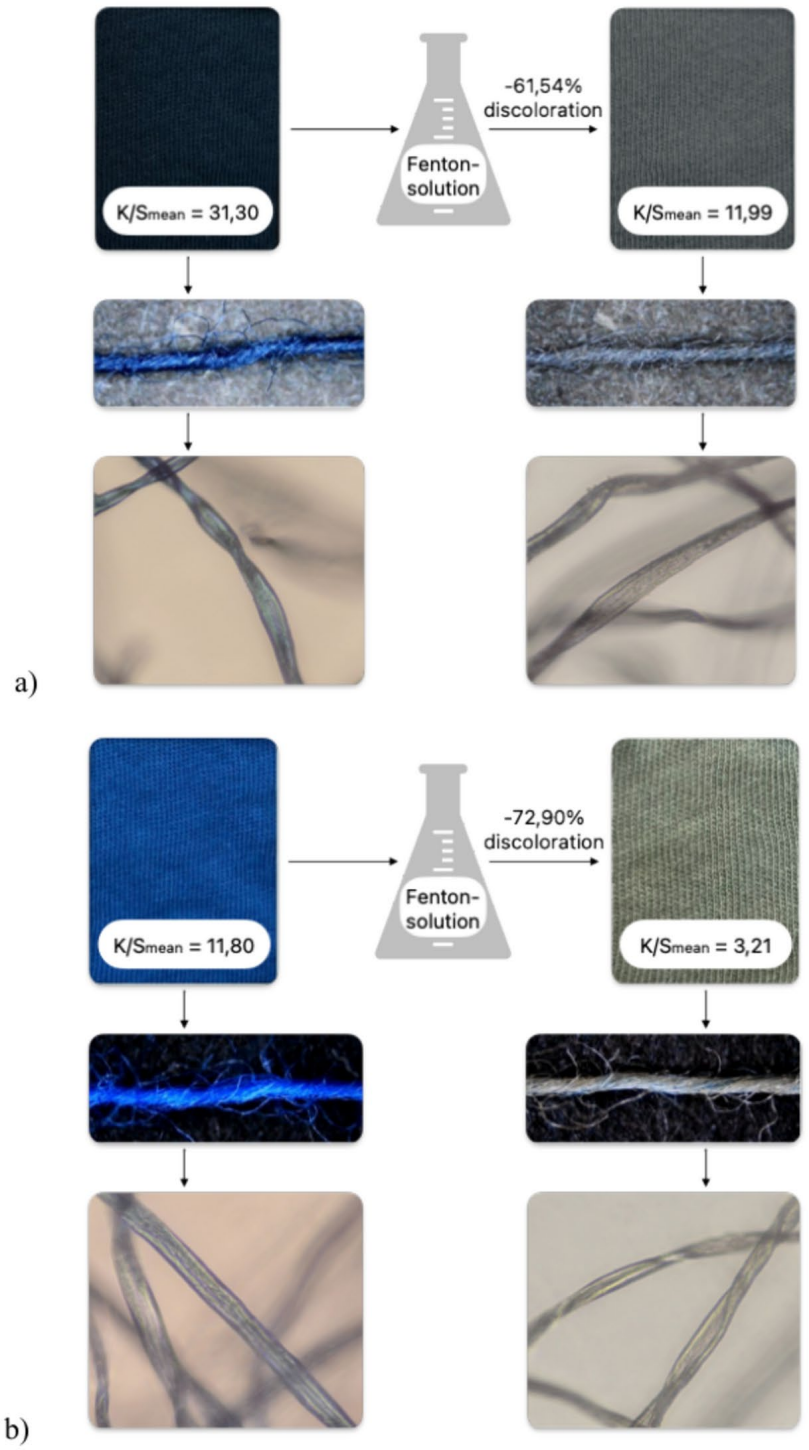
Discoloration of black-dyed cotton resulting in 61,54% color removal, considering the obtained mean K/S-values measured at λ_max_ = 600nm and a mean K/S_raw cotton_ = 0,072 (**a**) and discoloration of blue-dyed cotton resulting in 72,90% color removal, considering the obtained mean K/S-values measured at λ_max_ = 610nm and a mean K/S_raw cotton_ = 0,067 (**b**). Microscope images show the influence of the treatment on the scale of the cotton yarns and fibers.

Although the process did not lead to complete discoloration, the significant color stripping for both dyes show evidence for the effectiveness of the Fenton-based advanced oxidation processes. From the microscopy-images in Fig. 2, it is visible that the majority of the remaining color is situated towards the center of the yarn, where the fiber-density is higher, making it more difficult for the Fenton-reagents to reach the dye-molecules. In both cases also a change in chromaticity is visible (from black towards grey and from blue towards green), indicating partial degradation and thus changes in the molecular structure of the remaining dye, which leads to changes in absorption-behavior of the dye-molecules^45^.

A difference in sensitivity to the treatment is visible in the differing discoloration-efficiencies for the different dyes, which might be explained by the chemical structures of the dye-molecules; the black dye contains a di-azo-chromophore attached to several aromatic structures, while the blue dye consists of a less substituted anthraquinone ring which is more easily distorted^46^. Furthermore, the double binding-groups of the black dye can contribute to a more secure bond to the fibers, leading to enhanced stability.

**Fig. 3:**
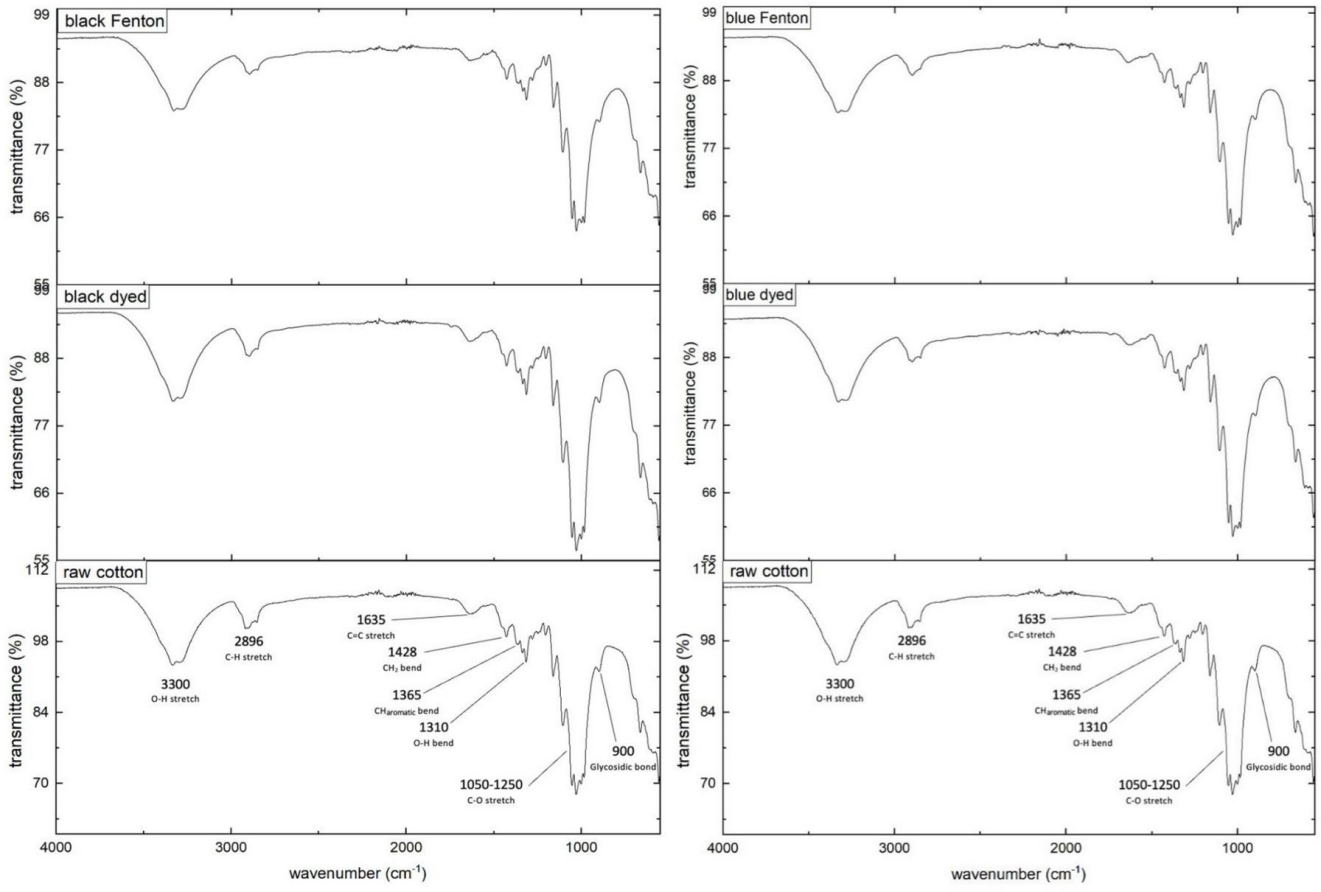
Stacked FTIR-spectra (transmittance-% versus wavenumber) of raw cotton, dyed cotton and Fenton-discolored cotton for the black (left) and blue dye (right).

In the FTIR-spectra given in Fig. 3, the most characteristic peaks of cotton are marked in the spectrum of raw cotton^47,48^. When comparing the spectra of the raw cotton, dyed cotton and Fenton-discolored cotton for the black and blue dye, the spectra do not show significantly noticeable changes or any additional peaks. Therefore, the IR-spectra seem to indicate that the main structure of the cotton is not altered due to the treatments. However, this spectroscopic method might obtain insufficient sensitivity to detect these supposed changes in the cellulose-structure, and thus additional thermal and mechanical analysis should be considered.

During the treatment, the mean pH was monitored; a drop from pH = 2.5 to pH = 2.2 was observed. According to literature, optimal pH for Fenton-mechanism lies between pH = 2.5–4^40,41^. Therefore, the pH in which these experiments occurred is rather low compared to the optimal range, and optimization of this parameter could further increase discoloration-efficiencies.

### Analysis of thermal and mechanical properties of discolored cotton fabric

**Fig. 4:**
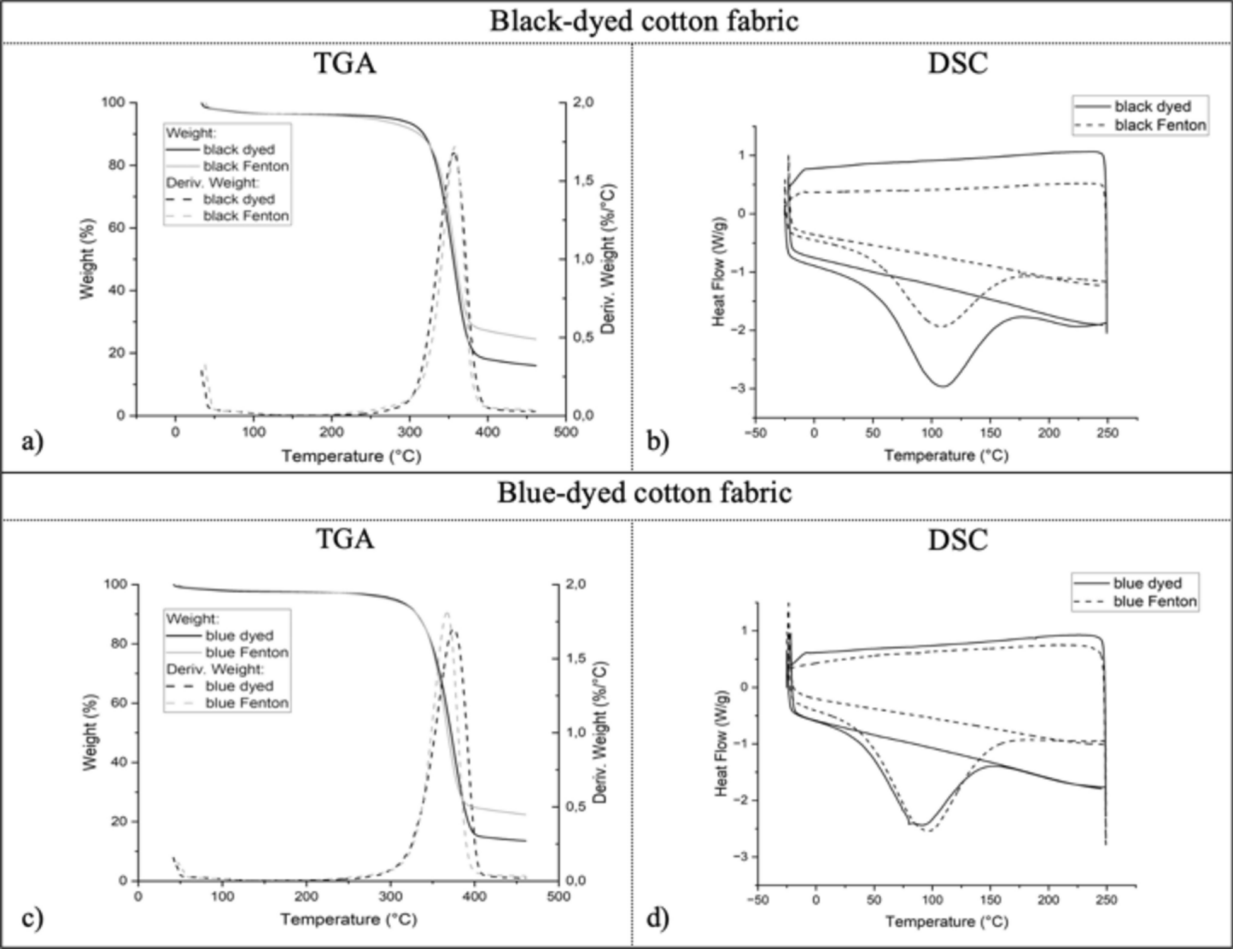
Plots of thermal analysis: TGA-plots of black-dyed cotton (**a**) and blue-dyed cotton (**c**) show the decrease in weight (%) (—) and weight derivative (---) during a temperature rise from 30 °C up to 500 °C for the dyed and Fenton-treated cotton. DSC-plots of black-dyed cotton (**b**) and blue-dyed cotton (**d**) show the heat-flow (W/g) during three heating/cooling cycles from − 25 up to 250 °C, for the dyed (—) and Fenton treated (---) cotton.

Overall, the thermal properties of the cotton before and after the discoloration-treatment resulted to be similar. From the TGA-plots of the black- and blue-dyed cotton before and after the Fenton-treatment in Fig. 4a, c, a small initial weight-loss between 30 and 110 °C is visible, which is caused by the vaporization of physically bound water^49^. The big decrease in weight between 220 and 450 °C can be attributed to decomposition of the cellulose-structure of the cotton^48^.

The mean decomposition temperatures during TGA of the black-dyed cotton before and after treatment (356.8 $\pm$ 2.5 °C and 358.1 $\pm$ 0.5 °C respectively) show no significant difference (*p* = 0.43). while those of the blue-dyed cotton (373.8 $\pm$ 1.1 °C and 365.4 $\pm$ 1.5 °C respectively) show a slight but significant difference (*p*  = 0.001), which might indicate a decrease in crystallinity after the Fenton-treatment since lower crystallinity can accelerate the degradation process and therefore reduce the thermal stability of the fiber^50^. However, further investigation of the crystallinity of the fibers is necessary to make more decisive conclusions on this matter.

In both cases, the mean weight residue after TGA increased significantly after Fenton-treatment (*p*  < 0.001) from 16.7 $\pm$ 0.4% to 25.1 $\pm$ 0.6% for the black-dyed cotton and from 13.3 $\pm$ 0.5% to 23.0 $\pm$ 0.4% for the blue-dyed cotton. This result indicates enhanced low-energy reactions of transforming the cellulose into char residue instead of volatilizing the cellulose into flammable gasses in high-energy reactions^51^ due to the Fenton-treatment.

The DSC-plots of the black- and blue-dyed cotton before and after the Fenton-treatment in Fig. 4b, d show an endotherm peak between 50 and 150 °C in the first cycle (heating from -25 up to 250 °C), which can be attributed to the elimination of physically bound water^49^ This peak is spread out over a broad range of temperatures because both water bound to the surface (which evaporates easily) and water in internal amorphous parts (which is bound by strong hydrogen-bonds and requires more heat to evaporate) are removed in this cycle^52^. This water is already removed in the first cycle, and therefore heating from − 25 up to 250 °C again in the third cycle occurs without endotherm peak of evaporation of water.

**Fig. 5:**
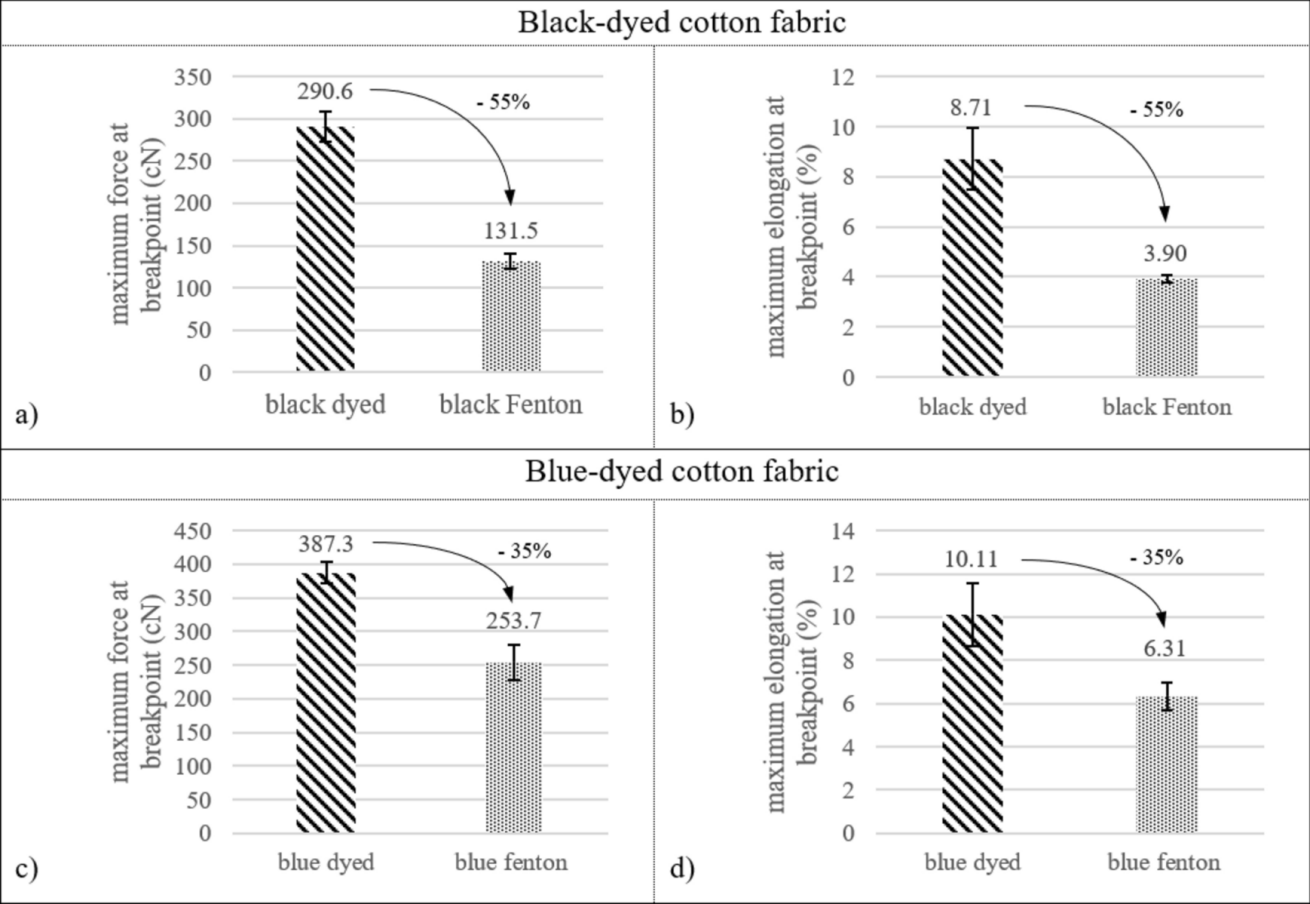
Results of mechanical analysis via tensile tests: average maximum force (cN) and elongation (%) at breakpoint of the unraveled yarn of untreated and Fenton-treated textiles of the black cotton (**a, b**) and blue cotton (**c, d**), with error-bars showing the standard deviation.

The Fenton-treatment also causes a significant reduction in tensile strength and elongation for both the black-dyed (-55% with *p* < 0.001 for both units) and the blue-dyed cotton (-35% with *p* < 0.001 for both units), as visible in Fig. 5. These reductions in mechanical properties might indicate a distortion of the cellulose-structure due to the treatment; plausible oxidation of cellulosic structure (for example at locations which are sensitive to oxidation, such as the C-O bonds of the alcohol functions or the glycosidic bond^53^) can lead to distortion of the crystalline regions of the cotton fibers, which is associated to reduction of tensile strength of the fibers^54^. Therefore, these results indicate that not only the dye-molecules but also the cellulosic structure is oxidized during the reaction, which leads to a decrease in crystallinity and strength of the fibers.

### Qualitative Analysis of mechanical recyclability

Opening the fabric and yarns into fibers allows for re-spinning into new fibers during mechanical recycling (also F2F mechanical recycling)^3^. Bigger amount of opened fabric and longer remaining fibers would enhance the mechanical recycling and the quality of the recycled yarns, and would allow reduced necessity of additional virgin cotton fibers^55^.

**Fig. 6:**
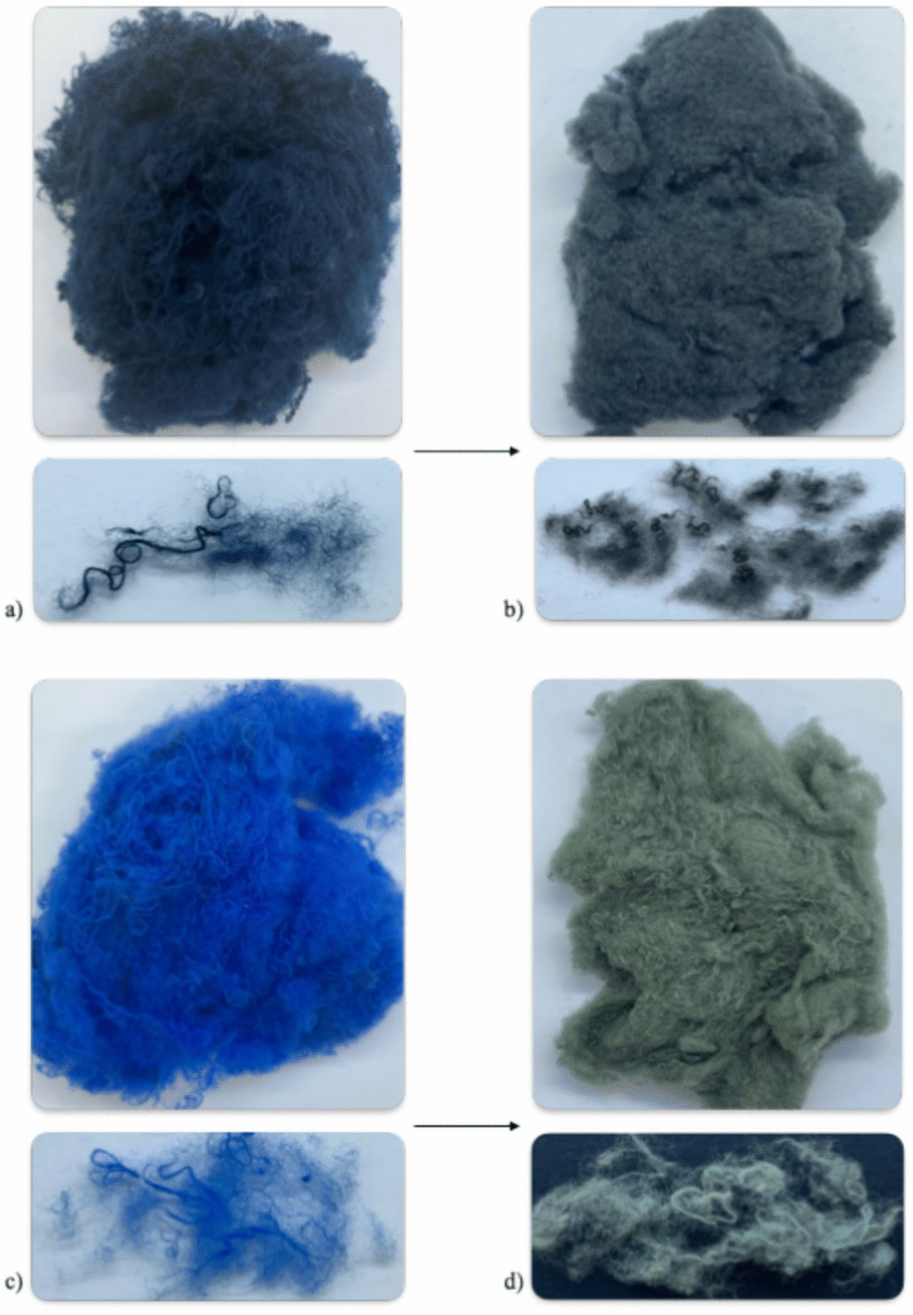
Images of opened fabric and remaining fibers of the black-dyed cotton (**a**), black Fenton-treated cotton (**b**), blue-dyed cotton (**c**) and blue Fenton-treated cotton (**d**).

An effort to analyze the shredded resultant of the dyed and Fenton-discolored cotton was performed by qualitatively comparing the amount of opened versus non-opened yarns and the length of these remaining non-opened yarns. Also, the length of the fibers of the opened fabric was qualitatively described and compared. The Fenton-treatment notably enhances the opening of the fabric in Fig. 6, resulting in increased amount of opened fabric and reduced length of the remaining non-opened yarns. This would facilitate the re-spinning during mechanical recycling. However, the shredding of the Fenton-treated cotton also results in shorter remaining opened fibers as is visible in the detailed images of Fig. 6, which can affect the quality of the recycled fiber negatively^55^. This enhanced opening but reduced length of remaining fibers is likely to be caused by the reduced tensile properties due to the oxidation of the cellulose during the treatment. How much reduction in fiber strength is allowed for good opening while maintaining sufficient tensile properties for re-spinning into new fibers needs to be investigated and optimized in future research articles.

When comparing the overall required amount of chemicals and temperature for an industrial process such as activated hydrogen peroxide discoloration, where 0.5–1M H_2_O_2_ at 70–90 °C and pH 10 for 60 to 180 min leads to discoloration percentages of 50–96%^9,10^, it is noticeable that the applied Fenton-treatments require lower amount of the same chemicals (0.28M H_2_O_2_) and lower amount of energy (at a temperature of 40 °C for 60 min) while achieving discoloration percentages of 62–73%. Lower amount of chemicals and energy will reduce the cost of the operation. However, the use of the metal catalyst increases cost of Fenton, and therefore the possibility of reuse of this metal catalyst should be investigated.

## Conclusion

The first applications of the chemical Fenton-oxidation for the color-stripping of reactive-dyed cotton in this feasibility study indicate promising discoloration percentages (61.54% for the black-dyed cotton and 72.90% for the blue-dyed cotton). This study was performed without complete optimization of all process-parameters (such as pH, reagent concentrations, temperature, time, liquor ratio’s), only a limited preliminary investigation of the influence of increasing Fenton-solution concentrations was performed to obtain the process-conditions for the upscaled main tests. In this study, lower amounts of the toxic and flammable chemicals and lower temperatures were required compared to the industrial alkaline hydrogen peroxide discoloration. However, more complete optimization via experimental design of the process-parameters is necessary to further increase discoloration-efficiencies, which might also allow milder conditions (lower temperature, lower concentrations, higher pH) while still achieving acceptable discoloration efficiencies.

The analysis of the resulting thermal and mechanical properties of the treated cotton indicates that besides the oxidation of the aromatic dye-molecules, also some oxidation of the cellulose-structure occurs, possibly causing reduction in crystallinity of the fibers and leading to reduced mechanical properties. Nonetheless, these reduced mechanical properties can also enhance opening of the cotton during shredding of the mechanical recycling. On the other hand, also shorter remaining fibers are obtained after shredding due to the oxidation of the cellulose, which could impact the quality of re-spun yarns negatively. In order to gain more insight into how the treatment affects the fiber-length and mechanical recyclability of the cotton fibers after shredding, further detailed research is suggested. Research for optimization of the process parameters might allow for milder conditions while still obtaining acceptable discoloration-efficiencies, which could cause less oxidation of the cellulosic chain structures and might lead to less reduction of mechanical properties of the discolored cotton.

Concerning the environmental impact of the treatment; besides the reduction of the required amount of the highly reactive and toxic chemical H_2_O_2_ (0.28M instead of 0.5–1M) and energy (40 °C for 60 min instead of 70–90 °C for 60–180 min) for this Fenton-based color-stripping treatment compared to the industrial alkaline hydrogen peroxide treatments^9,10^, the process should also aim for reduced carbon- and water-footprint and final toxicity. Therefore, future research towards the toxicity of the residues remaining in the Fenton-solution and on the fabric and thorough comparative studies on the influence of the treatments on the environment and textiles-materials between other current industrial discoloration processes and the Fenton-oxidation should be performed. Also, the economic aspects of this method and how to increase the economic appeal and industrial applicability should be investigated in order to develop a realistic alternative for industry.

## Supplementary Information

Supplementary Information 1.

Supplementary Information 2.

Supplementary Information 3.

Supplementary Information 4.

Supplementary Information 5.

Supplementary Information 6.

Supplementary Information 7.

Supplementary Information 8.

Supplementary Information 9.

## Data Availability

All data generated or analysed during this study is included in this published article.
