# Supplementary material for: Study on Fenton-based discoloration of reactive-dyed waste cotton prior to textile recycling
Source: Sci Rep. 2024 Oct 19;14:24536. doi: 10.1038/s41598-024-75450-w (PMC11489705; doi:10.1038/s41598-024-75450-w)
Supplement: Supplementary file 1 — Supplementary Information 1. [file 41598_2024_75450_MOESM1_ESM.docx]

**Supplementary document on** **Study on Fenton-based Discoloration of Reactive-dyed Waste Cotton prior to Textile Recycling**

**PRELIMINARY STUDY ON FENTON-SOLUTION CONDITIONS**

A preliminary study was performed to obtain the required parameters of Fenton reagents for the discoloration of waste cotton fabric, based on optimal conditions for discoloration of waste-water in literature, summarized in Table 1 in section 1 in the main study.

The influence of increasing concentration of the Fenton-solution on the efficiency of the discoloration of fabric was investigated; four different concentrations were tested, starting with the first treatment according to the optimal concentrations for Fenton-discoloration of dye-solution in Table 1 in section 1 in the main study. Three more treatment-parameters are decided by multiplying these optimal concentrations by 2, 4 and 8 (presented in Table S.1).

Table S.1: Four different conditions used in pre-tests, for a fabric of 5x5cm in 100 ml solution.

|  | [Fe^2+^] (mM) | [H_2_O_2_] (mM) | T (°C) |
| --- | --- | --- | --- |
| Fenton 1 | 3,5 | 70 | 40 |
| Fenton 2 | 7 | 140 | 40 |
| Fenton 3 | 14 | 280 | 40 |
| Fenton 4 | 28 | 560 | 40 |

1. **Method of discoloration of colored textile waste using Fenton-based advanced oxidation processes**

Cotton fabric-samples of 5x5cm are treated in 100 ml solution, according to the conditions in Table S.1, for both the black- and the blue-dyed fabric. For each dye, these pre-tests are repeated 4 times. The temperature is kept at 40°C by a warm-water-bath and the diffusion is ensured by magnetic mixing bars (stirring at 400 rpm). After the 60 minutes, the fabric-samples are rinsed and left to dry.

1. **Results and Discussion** **- Influence of Solution-concentration on Discoloration**

Bar-charts with mean K/S-values of the black- and blue-dyed cotton and the samples treated with increasing Fenton-solution concentrations (Fenton 1 – Fenton 4) are given in Figure S.1.

| Black-dyed cotton | Blue-dyed cotton |
| --- | --- |
| 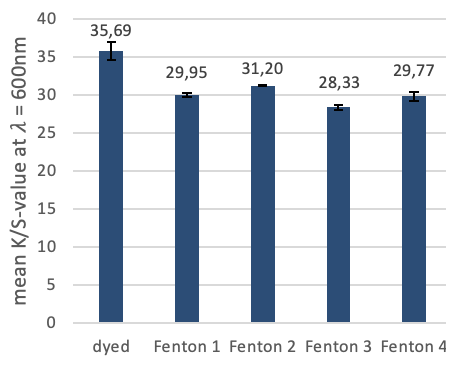 | 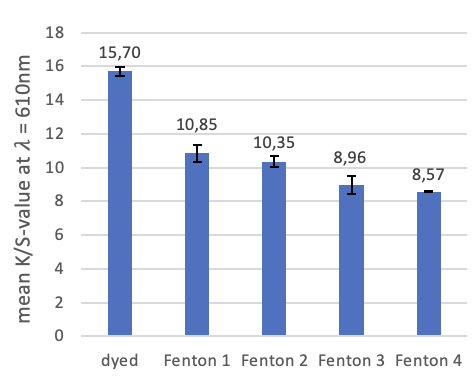 |

Figure S.1: mean K/S-values of the black-dyed (left) and blue-dyed (right) cotton before and after Fenton treatments with increasing concentrations according to Table S.1.

From these K/S-values, the associated discoloration percentages of the black- and blue-dyed cotton fabric can be calculated according to the equation in section 2.4 of the main study, and are given in Table S.2.

Table S.2: mean discoloration percentages of the black- and blue-dyed cotton for Fenton-treatments with increasing concentrations according to Table S.1.

|  | Fenton 1 | Fenton 2 | Fenton 3 | Fenton 4 |
| --- | --- | --- | --- | --- |
| Black-dye discoloration (%) | 15,89 | 12,39 | 20,46 | 16,40 |
| Blue-dye discoloration (%) | 30,62 | 33,76 | 42,71 | 45,15 |

To compare the K/S-results, statistical T-tests are performed between the K/S-values of the dyed and Fenton-treated samples, with all treatment-conditions from Table S.1 leading to significant discoloration, and the p-values given in Table S.3 and S.4.

The seemingly optimal Fenton-condition for discoloration of the black-dyed cotton (Fenton 3) is compared to the other treatments, to verify whether it gives significantly differing results compared to the other treatment-conditions, with these p-values also given in Table S.3.

Table S.3: p-values of t-tests between measured K/S-values of the black-dyed cotton before and after Fenton-treatments and p-values of t-tests between K/S-values of the seemingly optimal treatment (Fenton 3) and other Fenton-treatments, with all t-tests with $\alpha$ = 0,05 and n = 4.

|  | Fenton 1 | Fenton 2 | Fenton 3 | Fenton 4 |
| --- | --- | --- | --- | --- |
| Black-dyed fabric | 0,003 | 0,005 | 0,001 | 0,003 |
| Fenton 3 | 0,033 | 0,008 | / | 0,092 |

The seemingly optimal Fenton-condition for discoloration of the blue-dyed cotton (Fenton 4) is also compared to the other treatments, to verify whether it gives significantly differing results compared to the other treatment-conditions, with these p-values also given in Table S.4.

Table S.4: p-values of t-tests between measured K/S-values of the black-dyed cotton before and after Fenton-treatments and p-values of t-tests between K/S-values of the seemingly optimal treatment (Fenton 4) and other Fenton-treatments, with all t-tests with $\alpha$ = 0,05 and n = 4.

|  | Fenton 1 | Fenton 2 | Fenton 3 | Fenton 4 |
| --- | --- | --- | --- | --- |
| Blue-dyed fabric | < 0,001 | < 0,001 | < 0,001 | < 0,001 |
| Fenton 4 | 0,025 | 0,017 | 0,411 | / |

When comparing the seemingly optimal treatment for the black-dyed cotton (Fenton 3) to the other treatments by t-tests in Table S.3, only the results from Fenton 4 cannot be assumed significantly different from Fenton 3. However, with an eye on sustainability, it is more beneficial to consume a lower amount of chemicals. Since Fenton 4 requires double the amount of chemicals compared to treatment 3, further Fenton-treatments of the black-dyed fabric will be performed with the conditions of Fenton 3.

When comparing the seemingly optimal treatment for the blue-dyed cotton (Fenton 4) to the other treatments by t-tests in Table S.4, only the results from Fenton 3 cannot be assumed significantly different from treatment 4. Also here, with an eye on sustainability and consuming less chemicals, since no significant difference in result is obtained for the different concentrations further Fenton-treatments of the blue-dyed fabric will be performed with the conditions of Fenton 3 (considering Fenton 4 requires double the amount of chemicals compared to treatment 3). The decision to treat both the black- and blue-dyed fabric with the same Fenton-conditions for further experiments will also allow better comparison of the results of both fabrics.

In these preliminary studies, the obtained discoloration-percentages are lower (20,46% for black dye and 42,71% for the blue dye) compared to the upscaled measurements in the main study (61,54% for the black dye, 72,90% for the blue dye). This increased efficiency in the upscaled measurements could be due to two reasons; first of all, a pre-wetting step is added due to the rather hydrophobic nature of the raw-cotton used in this study, which enhances the reaction with the rather hydrophobic raw dyed cotton in the water-based Fenton-solution. Secondly, a rather old batch of H_2_O_2_-solution was used during the preliminary tests, while a new batch was used for the upscaled treatments. Since H_2_O_2_-solutions are relatively unstable, the old batch could likely have contained reduced amount of active H_2_O_2_ as is present in a newly ordered batch.
