# Supplementary figures and images for: Study on Fenton-based discoloration of reactive-dyed waste cotton prior to textile recycling
Source: Sci Rep. 2024 Oct 19;14:24536. doi: 10.1038/s41598-024-75450-w (PMC11489705; doi:10.1038/s41598-024-75450-w)

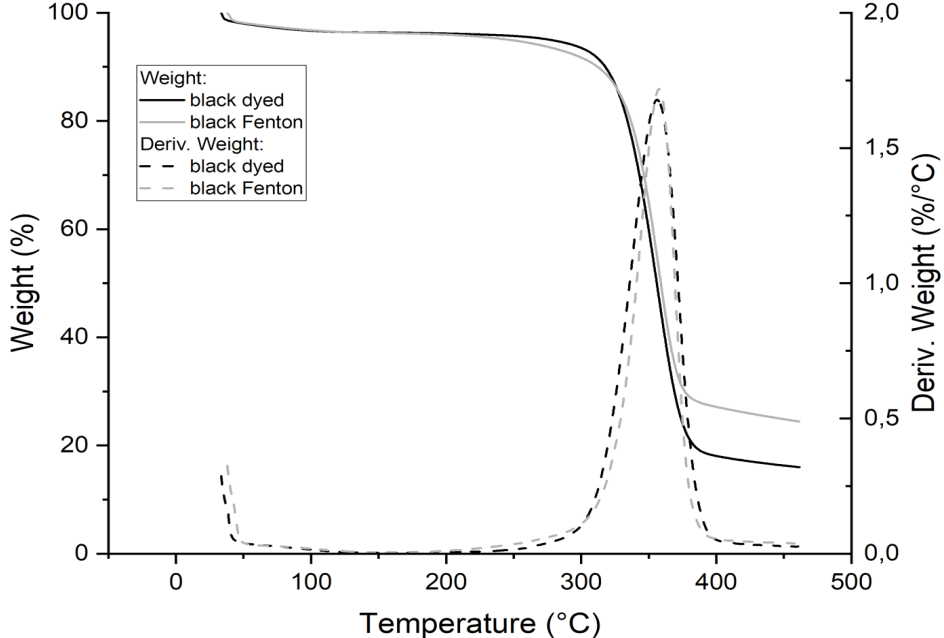

Supplement: Supplementary file 5 — Supplementary Information 5. [file 41598_2024_75450_MOESM5_ESM.pdf]

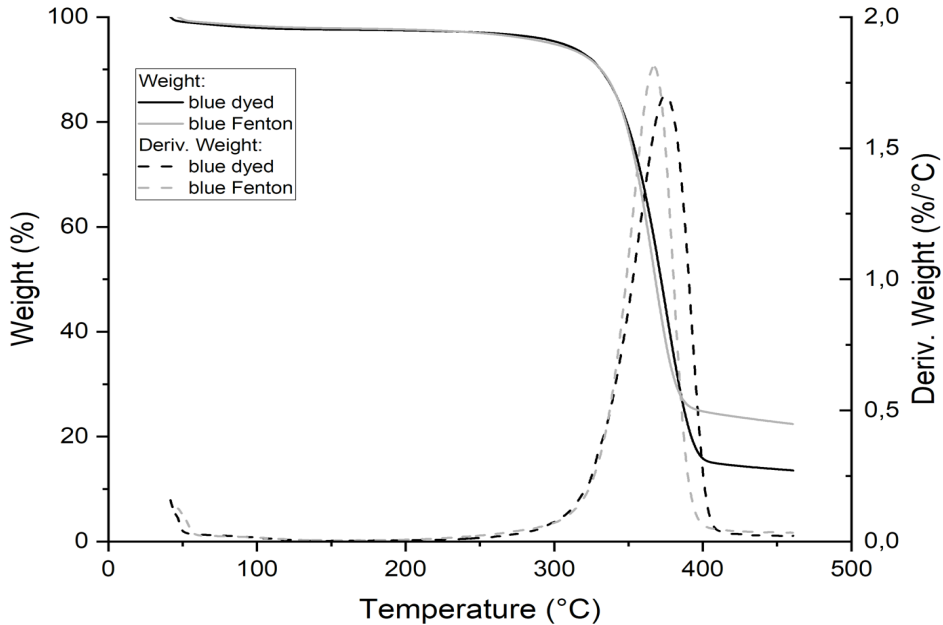

Supplement: Supplementary file 6 — Supplementary Information 6. [file 41598_2024_75450_MOESM6_ESM.pdf]

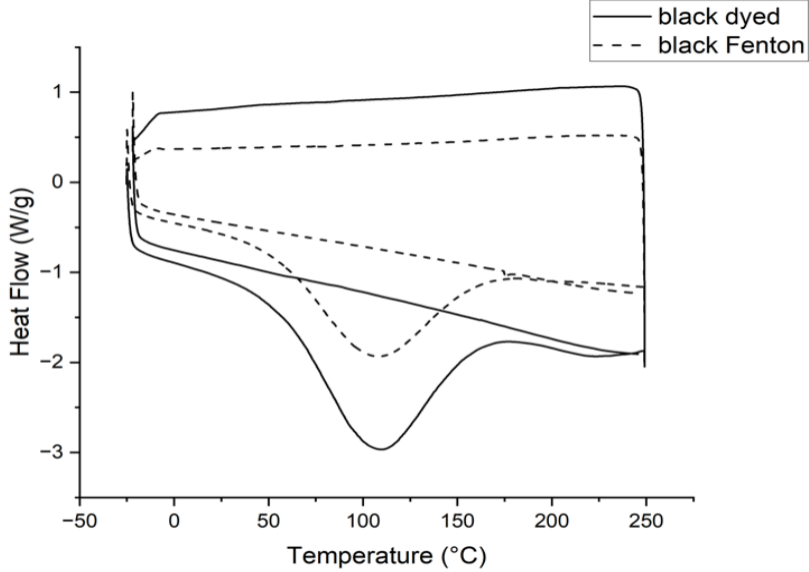

Supplement: Supplementary file 7 — Supplementary Information 7. [file 41598_2024_75450_MOESM7_ESM.pdf]

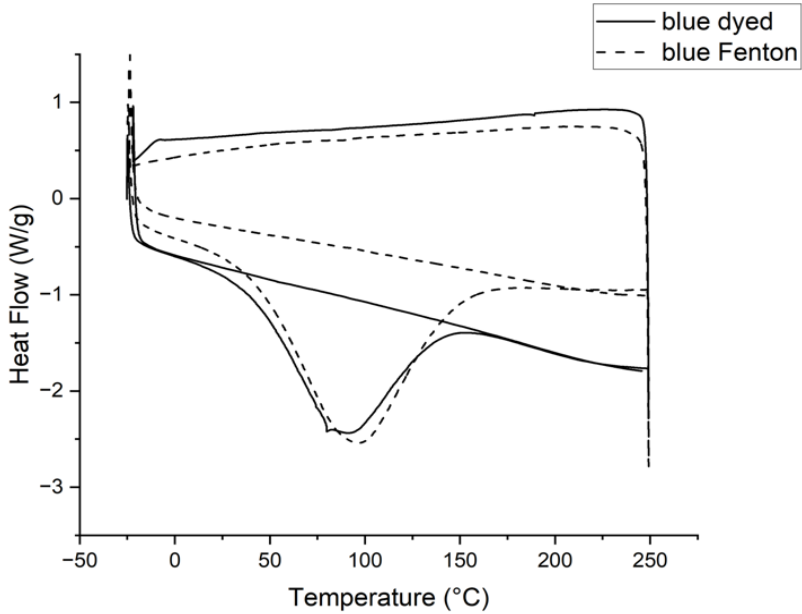

Supplement: Supplementary file 8 — Supplementary Information 8. [file 41598_2024_75450_MOESM8_ESM.pdf]
